# Dynamic neuromuscular stabilization versus traditional training: a randomized controlled trial comparing their effects on balance, posture, and quality of life in adolescents with increased thoracic kyphosis

**DOI:** 10.1186/s13102-025-01522-7

**Published:** 2026-02-02

**Authors:** Vahid Mazloum, Ali Honarvar, Zahra Jabarpour, Masoume Matin

**Affiliations:** 1 Department of Sport Injuries and Corrective Exercises, Ka.C, Islamic Azad University, Karaj, Iran; 2 Rehabilitation Research Center, Department of Physiotherapy, School of Rehabilitation Sciences, Iran University of Medical Sciences, Tehran, Iran

**Keywords:** Thoracic kyphosis, Postural balance, Quality of life, Exercise therapy, Spinal curvatures, Rehabilitation

## Abstract

**Background:**

Increased thoracic kyphosis (TK) in adolescence can negatively affect posture, balance, and quality of life (QoL). While conventional corrective exercises (CE) are commonly used, Dynamic Neuromuscular Stabilization (DNS), based on developmental kinesiology, has shown potential as a targeted intervention. This study aims to compare the effects of DNS and conventional CE on TK angle, dynamic balance, and QoL in adolescent boys with increased TK.

**Methods:**

In this single-blind randomized controlled trial, 60 male adolescents aged 14–16 years with increased TK were randomly assigned to three groups: DNS, conventional CE, or control. The DNS and CE group completed six sessions per week (three supervised and three home-based) for 8 weeks and control group continues their normal life. TK angle was measured using a Spinal Mouse device, dynamic balance was assessed with the Y-Balance Test, and QoL was evaluated using the SF-36 questionnaire.

**Results:**

Analysis revealed that both the DNS and conventional exercise (CE) groups showed statistically significant improvements in all measured outcomes, including thoracic kyphosis (TK) angle, dynamic balance, and quality of life (QoL) compared to the control group (p < 0.05). Furthermore, while the DNS group demonstrated significantly greater improvements than the CE group in TK angle and dynamic balance (p < 0.05), the difference in QoL between the two intervention groups was not statistically significant.

**Conclusion:**

DNS exercises were more effective than conventional CE in improving posture, balance, and QoL in adolescents with increased TK.

**Trial registration:**

This trial was registered in the Iranian Registry of Clinical Trials (IRCT) under the registration code IRCT20240907062968N5. Registration Date: 13/05/2025.

**Supplementary Information:**

The online version contains supplementary material available at 10.1186/s13102-025-01522-7.

## Introduction

Increased thoracic kyphosis (TK) is a common postural condition that often begins during adolescence, a critical period of rapid growth and physical development, and may progressively worsen with age [1]. Recently, adolescents have become more susceptible to postural disorders, including increased TK, likely due to the widespread use of digital devices such as smartphones and personal computers [2]. Currently, approximately 25% of adolescents exhibit increased TK, highlighting the rising prevalence of this condition [3].

During rapid growth phases, insufficient physical activity and poor daily habits can weaken trunk muscles, leading to spinal misalignment [4]. Moreover, if spinal deformities are not addressed during adolescence, they may result in persistent issues in adulthood [5]. In adults, increased TK is often associated with musculoskeletal, neuromuscular, and sensory impairments such as weakened spinal extensors, altered spinal curvature, pain, shoulder dysfunction, and balance deficits [6, 7].

Moreover, biomechanical changes such as a forward shift in the center of gravity and increased postural sway can disrupt balance and raise the risk of falls [7]. Therefore, it’s especially important to pay close attention to increased TK, particularly during adolescence. Various exercise intervention have been reported for increased TK, and corrective exercise (CE) also was reported as an intervention method attributed to improve spinal posture, balance, and well-being [8]. CE programs, including resistance training, stretching, and postural education, have demonstrated effectiveness in improving thoracic posture and reducing the kyphosis angle [9].

Recently, Dynamic Neuromuscular Stabilization (DNS) exercises have gained attention as a promising method for individuals with musculoskeletal and neurological conditions. By activating core muscles and engaging the integrated spinal stabilization system (ISSS), DNS enhances spinal stability, reduces pain, and improves functional movement [10].

However, no study has yet examined the effects of DNS exercises on TK in adolescents. Therefore, this study is the first to investigate the impact of these exercises on increased TK and to compare their effectiveness with conventional CE.

## Methods

### Study design

This study was a single-blind randomized controlled trial with a pretest-posttest design conducted over 8 weeks. The trial was registered in the Iranian Registry of Clinical Trials (IRCT) under registration code IRCT20240907062968N5. Participants were randomly assigned to one of three groups: (1) a conventional CE group (2), DNS group, or (3) a control group. The study adhered to the ethical principles outlined in the 1964 Helsinki Declaration and its subsequent amendments. This study followed the CONSORT guidelines for reporting randomized controlled trials.

The Inclusion criteria for the study were: (1) increased TK, and (2) the ability to sit, stand, walk, and perform daily activities independently. Exclusion criteria included: (1) history of spinal surgery (2), failure to complete the intervention (3), use of other treatment methods during the study (4), injury or trauma during the intervention period (5), exacerbation of pain or disability due to exercises, and (6) unwillingness to continue participation. Possible adverse events (such as joint pain, muscle soreness, or injury) were monitored throughout the intervention period. Participants were asked at each session if they experienced any discomfort, and all reports were recorded by the research staff.

### Participants

For this study, 60 adolescent boys aged 14 to 16 years with increased TK were purposefully selected from schools in Alborz province. The participants were randomly assigned into three groups of 20. Prior to enrollment, all participants were fully informed about the study’s objectives and procedures and provided written informed consent before initial measurements.

### Sample size

The required sample size was estimated using G*Power software based on previous studies, considering a power of 0.80, an alpha level of 0.05, and a medium effect size (f = 0.25, equivalent to Cohen’s d = 0.6). This analysis determined that 20 participants per group were sufficient to detect significant differences between groups, resulting in a total of 60 participants.

### Randomization and blinding

Randomization was performed using a computer-generated random number sequence to ensure unbiased allocation of participants to the intervention groups. To maintain allocation concealment, the group assignments were placed in sequentially numbered, sealed, opaque envelopes prepared by an independent researcher who was not involved in participant recruitment, assessment, or intervention. These envelopes were opened only after each participant completed baseline measurements. Due to the nature of the exercise interventions, participants knew their group assignments; however, outcome assessors and statistical analysts were blinded to reducing the risk of measurement and interpretation bias.

### Outcome measures

#### Y-balance test

**Fig. 1:**
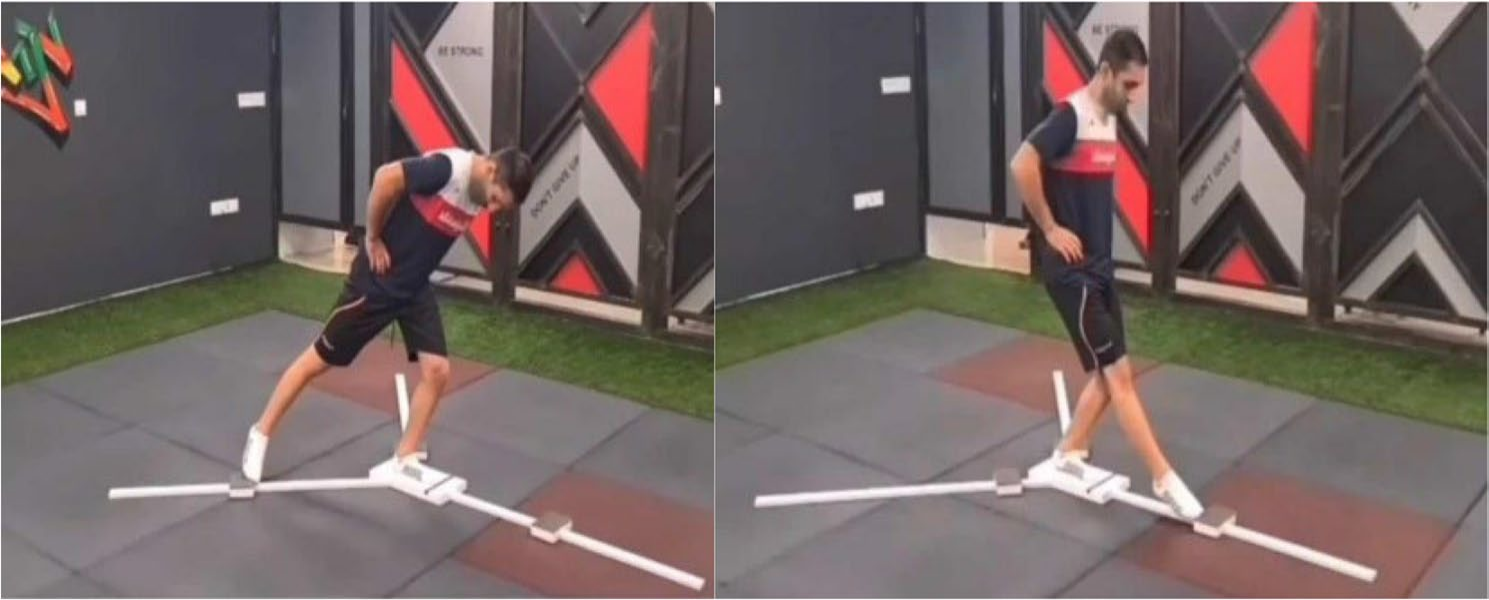
Y-balance test

Dynamic balance was evaluated using the Y-Balance Test, which has demonstrated high intra-rater reliability in previous studies [11] (Figure.1). Participants first watched an instructional video to become familiar with the proper technique. To minimize the influence of learning effects, each participant practiced the test by performing three movements in each direction—anterior, posteromedial, and posterolateral. Subsequently, they completed three full test trials, with a two-minute rest interval between each. A trial was deemed invalid and repeated if the participant: [1] was unable to maintain single-leg stance [2], touched the floor with the reaching leg due to instability [3], shifted the stance leg, or [4] failed to return to the initial position. The average of the three successful trials in each direction was calculated, normalized to limb length, and expressed as a percentage. Limb length was determined with the participant in a supine position, measuring from the anterior superior iliac spine to the most distal point of the medial malleolus after ensuring proper pelvic alignment [12]. Assessments were conducted by the same experienced assessor who was blinded to group allocation. The assessor received prior training in the use of Y-Balance Test.

### Thoracic kyphosis angle

**Fig. 2:**
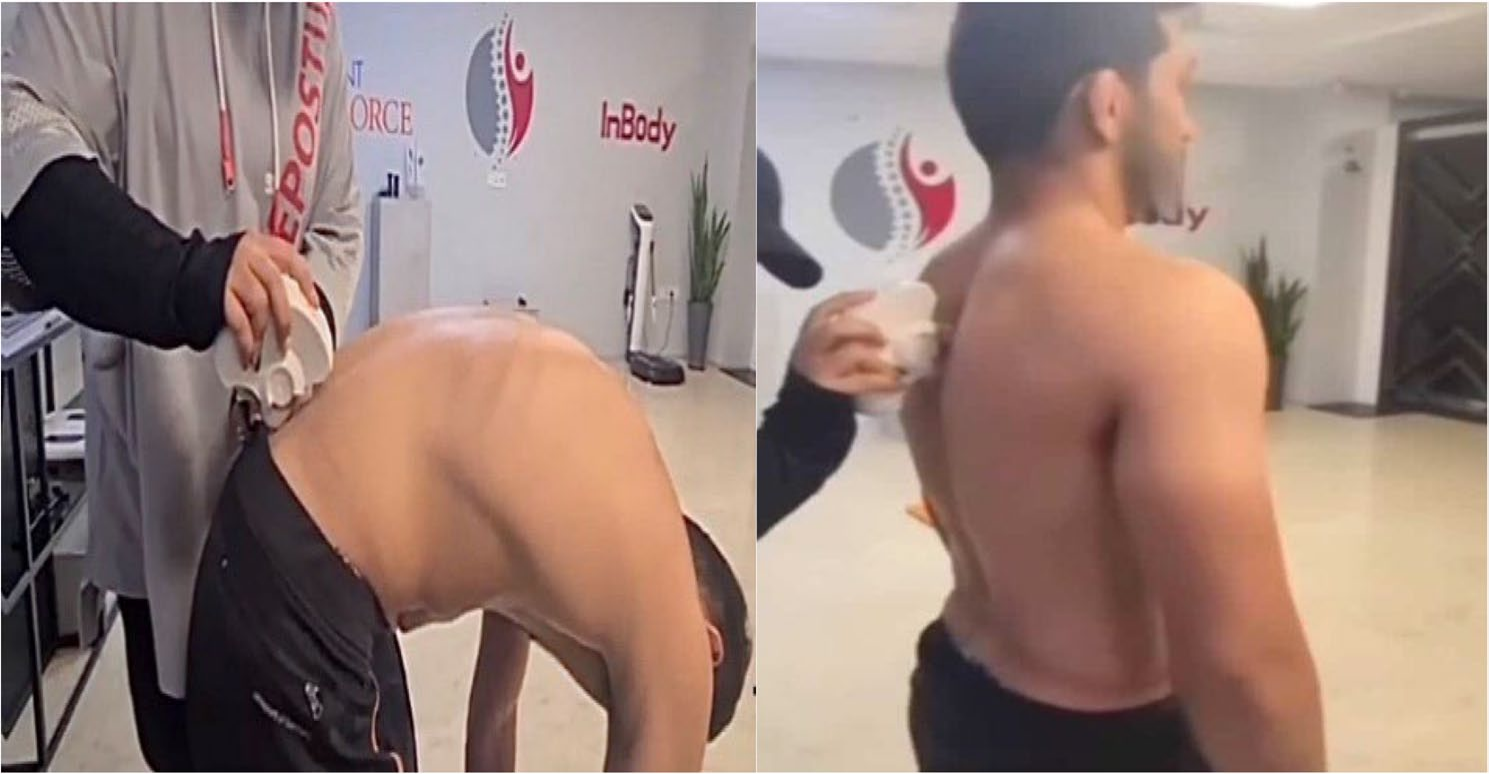
Measurement of TK Angle

The Spinal Mouse is used to assess the TK angle, which is crucial in evaluating spinal alignment and posture and the reliability and validity of the Spinal Mouse system for measuring spinal curvature parameters, including TK, have been confirmed in prior research [13] (figure.2). During the measurement procedure, participants are instructed to remove their upper body clothing and assume a natural standing posture at a predetermined location. The assessor then marks the spinous processes of the seventh cervical vertebra(C7) and the second sacral vertebra (S2) as the initial and final landmarks, respectively, using a marker. The Spinal Mouse roller is positioned on the initial landmark and moved toward the final landmark along the spinous processes of the vertebrae. TK was calculated based on the thoracic curve (e.g., from T1 to T12) extracted from the scan between C7 and S2. The data from the Spinal Mouse are transmitted via radio waves to a computer, where the spinal curvature angles are calculated and recorded [14].

### Quality of life (QoL)

The Short Form 36 (SF-36) questionnaire was used to assess the QoL in this study. The SF-36 includes eight subscales; each composed of two to ten questions. These subscales are as follows: physical functioning, physical role limitations, bodily pain, general health, vitality, social functioning, emotional role limitations, and mental health. The integration of these subscales forms two broad categories: physical health (including physical functioning, physical role limitations, bodily pain, and general health) and mental health (including vitality, social functioning, emotional role limitations, and mental health).

This questionnaire can be completed either through self-report by the individual or through an interview conducted by the researcher. The reliability and validity of the SF-36 have been extensively evaluated in both healthy individuals and patients with various health conditions. Wu et. el [15] reported a reliability coefficient of 0.85 and validated the tool against the Nottingham Health Profile, confirming its strong construct validity across all subscales [15].

### Interventions

#### DNS protocol

The DNS exercise protocol involved six sessions per week (three supervised sessions and three home-based sessions) for 8 weeks (Table 1). In the supervised sessions, a physiotherapist provided both verbal and manual guidance for technique correction. Regarding the home-based exercises, participants were instructed on the correct techniques and subsequently asked to record videos and keep logs of their sessions. These materials were reviewed weekly by the research team to monitor adherence and ensure proper execution. Special attention was given to breathing patterns, particularly inhalation and exhalation. Participants were encouraged to properly activate the abdominal muscles and rib cage during exercises.

The home-based exercise program was similar to the supervised sessions, with participants receiving necessary training during the supervised sessions before performing exercises at home. Additionally, participants agreed not to engage in any other physical activities during the 6-week trial. Both supervised and home-based exercises were conducted according to the specified protocol, six times a week [16].

**Table 1:** DNS protocol

Week	Exercise Type	Sets	Repetitions & Rest Time
Week 1	Basic DNS movements	3 sets	10 reps, 2s inhale, 4s exhale, 60–90 s rest
Week 2	Basic DNS movements	3 sets	10 reps, 2s inhale, 4s exhale, 60–90 s rest
Week 3	Breathing exercises	3 sets	10 reps, 2s inhale, 4s exhale, 60–90 s rest
Week 4	Support on elbows, back lying, crawling position	3 sets	15 reps, 3s inhale, 6s exhale, 120–150 s rest
Week 5	Rolling pattern, back lying, crawling support	3 sets	15 reps, 3s inhale, 6s exhale, 120–150 s rest
Week 6	Quadruped, side plank, seated support	3 sets	20 reps, 3s inhale, 6s exhale, 120–150 s rest
Week 7	Crawling, sitting, side plank, elevated position	3 sets	20 reps, 3s inhale, 6s exhale, 120–150 s rest
Week 8	Kneeling, bear position, squat, standing	3 sets	20 reps, 3s inhale, 6s exhale, 120–150 s rest

### Routine CE protocol

The aim of the designed program was to stretch the shortened muscles in the anterior chest and strengthen the posterior muscles. The exercise program consisted of six sessions per week (three supervised sessions and three home-based sessions) for 8 weeks, each lasting between 30 and 45 min, under the supervision of the researchers. The home-based sessions were monitored like DNS group. Seven different exercises were prescribed for the participants, which included a combination of strength training, stretching, and functional activities. The structure and content of the program were developed based on previous research and established CE protocols (Table 2) [4, 17].

**Table 2:** Routine corrective exercises protocol

Week	Exercise	Sets	Repetitions
Week 1	Scapular Retraction, Shoulder Shrugs, Shoulder Abduction, External Shoulder Rotation, Chest Stretch, Superman	3 sets	10 reps
Week 2	Scapular Retraction, Shoulder Shrugs, Shoulder Abduction, External Shoulder Rotation, Chest Stretch, Superman	3 sets	10 reps
Week 3	Scapular Retraction, Shoulder Shrugs, Shoulder Abduction, External Shoulder Rotation, Chest Stretch, Superman	3 sets	15 reps
Week 4	Scapular Retraction, Shoulder Shrugs, Shoulder Abduction, External Shoulder Rotation, Chest Stretch, Superman	3 sets	15 reps
Week 5	Scapular Retraction, Shoulder Shrugs, Shoulder Abduction, External Shoulder Rotation, Chest Stretch, Superman	3 sets	20 reps
Week 6	Scapular Retraction, Shoulder Shrugs, Shoulder Abduction, External Shoulder Rotation, Chest Stretch, Superman	3 sets	20 reps
Week 7	Scapular Retraction, Shoulder Shrugs, Shoulder Abduction, External Shoulder Rotation, Chest Stretch, Superman	3 sets	25 reps
Week 8	Scapular Retraction, Shoulder Shrugs, Shoulder Abduction, External Shoulder Rotation, Chest Stretch, Superman	3 sets	25 reps

### Control group

Participants in the control group did not receive any specific intervention and were instructed to continue their normal daily routines without participating in any additional exercise or therapeutic programs during the study period.

### Statistical analysis

All statistical analyses were conducted using SPSS software (version 21.0; IBM Corp., Armonk, NY, USA). Descriptive statistics, including means and standard deviations, were calculated to summarize participant characteristics and outcome measures. The normality of data distribution was assessed using the Shapiro–Wilk test.

To compare the effects of the intervention between groups while controlling baseline differences, analysis of covariance (ANCOVA) was performed using pre-test scores as covariates. The significance level of *p* < 0.05 was considered statistically significant for all analyses. Only participants who completed both the intervention and the final assessments were included in the analysis (per-protocol analysis). Each participant was analyzed in the group to which they were originally assigned.

## Results

Of the 80 participants, 63 met the inclusion criteria and were randomly allocated to one of three groups, with 20 participants assigned to each group. One additional participant was allocated to each group in anticipation of potential dropouts. During the intervention period, one participant from the DNS group and one from the control group withdrew due to absence from training sessions or outcome measurements. These individuals were replaced by the pre-assigned extra participants. The addition of one extra participant per group did not compromise the integrity of the randomization process. Ultimately, 61 participants completed the study, and 60 were included in the final analysis (DNS: *n* = 20; CE: *n* = 20; Control: *n* = 20).

There were no significant differences among the groups at baseline (*p* > 0.05) (Table 3). Post-hoc comparisons using the Bonferroni correction revealed that both the DNS and CE groups showed significantly greater improvements than the control group in all outcomes (*p* < 0.01). Furthermore, the DNS group demonstrated significantly greater improvement than the CE group in TK angle, and dynamic balance (*p* < 0.01). Although the DNS group demonstrated slightly greater improvements in QoL than the CE group, the difference between-groups was small (Cohen’s *d* = 0.13) and not statistically significant. Summary of Outcome Measures are presented in (Table 4).

**Table 3:** Baseline characteristics of participants in the three groups (Mean ± SD)

Variable	Conventional Exercises (*n* = 20)	DNS Exercises (*n* = 20)	Control (*n* = 20)	*p*-value
Age (years)	15.56 ± 0.59	15.61 ± 0.60	15.58 ± 0.61	0.92
Height (cm)	155.5 ± 7.2	155.8 ± 7.0	155.3 ± 7.1	0.95
Weight (kg)	56.2 ± 13.4	56.6 ± 13.1	56.4 ± 13.2	0.97

**Table 4:** Summary of outcome measures (Mean ± SD)

Assessment Criterion	Assessment Time	Group	Mean ± SD	Assessment Time	Mean ± SD	*P*-value	Effect sizes
Kyphosis angle	Pre-test	CE	50.15 **±** 4.76	Post-test	45.15 **±** 4.99		
		DNS	47.10 **±** 3.54		38.15 **±** 3.72	*p* < 0.001	0.840
		Control	52.9 **±** 4.98		52.85 **±** 4.86		
Y-balance	Pre-test	CE	64.13 **±** 6	Post-test	70.55 **±** 6.41		
		DNS	66.68 **±** 7.67		76.08 **±** 8.17	*p* < 0.001	0.932
		Control	67.18 **±** 8.17		67.40 **±** 8.49		
Quality of life	Pre-test	CE	54.47 **±** 9.65	Post-test	66.01 **±** 10.34		
		DNS	63.08 **±** 9.76		74.28 **±** 8.06	*p* < 0.001	0.357
		Control	62.36 **±** 8.42		68.08 **±** 8.80		

## Discussion

The present study aimed to compare the effects of DNS exercises and conventional CE on TK angle, dynamic balance, and QoL in adolescents with increased TK. The findings revealed that while both intervention groups experienced significant improvements compared to the control group, the DNS group showed greater outcomes across all measured variables. These results suggest that DNS may be a more effective therapeutic approach for improving postural alignment, dynamic stability, and perceived QoL in this population.

CE programs target maintaining spinal alignment under both static and dynamic conditions and enhancing musculoskeletal control [4]. Consistent with previous research, participants in the CE group experienced significant reductions in TK angle and improvements in dynamic balance compared to controls. These benefits are likely attributed to activation and stretching of thoracic extensor muscles and improved proprioceptive input, which collectively facilitate postural correction [18]. These exercises are commonly used to enhance the flexibility of contractile tissues and to facilitate proper postural control. In adult males, interventions involving stretching and strengthening of the thoracic region have been reported to alleviate pain and lead to significant improvements in TK [19].

Consistent with the findings of the present study, previous research involving a sample of 62 adolescents with increased TK reported that a multi-component CE program, which included postural awareness training, led to a significant reduction in the kyphosis angle and marked improvements in dynamic balance [4]. Similarly, several studies have reported the same beneficial results after corrective protocols on increased TK in various age ranges [7, 8, 20]. In addition, Stretching activates mechanoreceptors located in muscles and joints, which in turn can enhance proprioceptive awareness, improve muscle coordination, and support more efficient neuromuscular function [9]. Considering the use of stretching exercises in the routine CE group in this study, this may explain the significant improvements observed in this group compared to the control group.

Furthermore, the DNS approach, grounded in developmental kinesiology principles, integrates diaphragmatic breathing with joint stabilization and segmental control in various developmental postures [21]. Correct breathing techniques not only improve respiratory efficiency but also contribute significantly to spinal stability [22]. For rehabilitation programs targeting the movement system to be effective, it is essential to retrain dysfunctional breathing patterns through structured breathing exercises across various postural conditions [23, 24]. By promoting coordinated muscle activation in these positions, DNS enhances motor control and supports functional stability [25]. This comprehensive strategy may underlie the significant improvements in TK angle, dynamic balance, and QoL observed in the DNS group after eight weeks of training.

Supporting these findings, A study by Afsari et el.[25] demonstrates that implementing six week of DNS training can significantly improve chest mobility, lower chest mobility, upright sitting height, and QOL in obese women [25]. Additionally, systematic reviews emphasize the positive impact of DNS exercises on rehabilitation outcomes in musculoskeletal and neurological conditions, highlighting the role of core muscle activation and ISSS [10].

Furthermore, the findings of this study are supported by previous systematic reviews that emphasize the effectiveness of exercise-based interventions in reducing TK. Jenkins et al. [26] reported that short-term structured exercise programs had a large effect in reducing TK in younger adults, while the effects in older adults were small to moderate. They also noted that combining exercise with manual therapy did not enhance outcomes in older populations [26]. Similarly, Dimitrijević et al. [27] found that CE significantly reduced the kyphosis angle, regardless of age group [27]. Additionally, Ponzano et al. [28] demonstrated that structured exercise programs improved thoracic curvature, spinal extensor strength, and QoL in older adults [28]. Compared to these findings, our study also confirms the beneficial effects of CE, but highlights that DNS, which incorporates postural reflex patterns and core stabilization. However, what remains inconsistent across studies is the effectiveness of such interventions in different age groups. Therefore, further research is needed to determine whether DNS exercises yield similar benefits in middle-aged and older adults as they do in younger individuals.

However, variations in intervention effectiveness across age groups remain unclear, warranting further research. Future studies should examine whether DNS yields comparable benefits in middle-aged and older adults as observed in younger individuals.

To the best of our knowledge, it is the first to compare DNS exercises with CE in adolescents with increased TK. A review of existing literature did not reveal any previous studies that directly examined the effects of DNS in comparison with other therapeutic approaches for kyphosis correction. This underscores the novelty of the current work and its potential contribution to clinical practice and rehabilitation science. However, certain limitations should be considered. First, the study population consisted solely of male adolescents from a single geographic area, limiting the generalizability of the results to females, other age groups, or individuals with more severe deformities. The absence of direct measurements of upper limb and trunk muscle strength limits a more detailed understanding of the mechanisms behind the observed improvements. Additionally, the lack of follow-up assessments prevents conclusions about the long-term effects of the interventions. Lastly, repeated administration of the Y-Balance Test could have introduced learning effects, especially in younger participants who may show rapid motor learning.

Future research should incorporate strength assessments and long-term follow-up at six or twelve months to evaluate sustained effects. Inclusion of female participants and broader demographic samples would enhance external validity. Comparative studies involving DNS and other neuromuscular or postural training modalities are also recommended to contextualize DNS’s role in clinical practice. Finally, while the DNS group’s home-based sessions were monitored weekly, future interventions might benefit from more frequent supervision to improve adherence and consistency.

## Conclusion

This study showed that both DNS and CE can improve TK, dynamic balance, and QoL in adolescents with increased TK. However, DNS led to greater improvements across all areas, highlighting its potential as a more effective treatment option. Because DNS combines muscle coordination, breathing, and proper posture, it might offer unique benefits that traditional exercises don’t fully address. While these results are promising, further studies with longer follow-ups and more detailed assessments are needed to better understand how DNS works and its long-term effects.

## Supplementary Information

Supplementary Material 1.

Supplementary Material 2.

## Data Availability

Due to ethical restrictions, the datasets generated during the current study are available from the corresponding author on reasonable request.

## Abbreviations

DNS: Dynamic neuromuscular stabilization
Tk: Thoracic kyphosis
QoL: Quality of life
ISSS: Integrated spinal stabilization system
IRCT: Iranian registry of clinical trials
CE: Corrective exercises

## References

[1] Yang S, Yi YG, Chang MC. The effectiveness of exercise programs in adolescents with thoracic kyphosis: A narrative review. Healthcare. 2024;12(15):1503. 39120206 10.3390/healthcare12151503 PMC11312307

[2] Ersen O, Yuzuguldu U, Karadamar OL, Basak AM, Ege T. Health-related quality of life of patients with postural kyphosis compared to spinal deformities in adolescence: A cross-sectional study. Turk Neurosurg. 2023;34:475–9. 10.5137/1019-5149.JTN.43440-23.2 38650564

[3] González-Gálvez N, Vaquero-Cristóbal R, López-Vivancos A, Albaladejo-Saura M, Marcos-Pardo PJ. Back pain related with age, anthropometric variables, sagittal spinal curvatures, hamstring extensibility, physical activity and health related quality of life in male and female high school students. Int J Environ Res Public Health. 2020;17(19):7293. 33036288 10.3390/ijerph17197293 PMC7579385

[4] Elpeze G, Usgu G, editors. The effect of a comprehensive corrective exercise program on kyphosis angle and balance in kyphotic adolescents. Healthcare: MDPI; 2022. 10.3390/healthcare10122478 PMC9778671 36554002

[5] Tarasi Z, Rajabi R, Minoonejad H, Shahrbanian S. The effect of spine strengthening exercises and posture training on functional thoracic hyper kyphosis in young individuals. J Adv Med Biomedical Res. 2019;27(121):23–31.

[6] Benedetti MG, Berti L, Presti C, Frizziero A, Giannini S. Effects of an adapted physical activity program in a group of elderly subjects with flexed posture: clinical and instrumental assessment. J Neuroeng Rehabil. 2008;5:1–11. 19032751 10.1186/1743-0003-5-32 PMC2613395

[7] Eftekhari E, Sheikhhoseini R, Salahzadeh Z, Dadfar M. Effects of telerehabilitation-based respiratory and corrective exercises among the elderly with thoracic hyper-kyphosis: a clinical trial. BMC Geriatr. 2024;24(1):234. 38448857 10.1186/s12877-024-04779-8 PMC10918978

[8] Moon H, Lee S-K, Kim W-M, Seo Y-G. Effects of exercise on cervical muscle strength and cross-sectional area in patients with thoracic hyperkyphosis and chronic cervical pain. Sci Rep. 2021;11(1):3827. 33589667 10.1038/s41598-021-83344-4 PMC7884681

[9] Kamali F, Shirazi SA, Ebrahimi S, Mirshamsi M, Ghanbari A. Comparison of manual therapy and exercise therapy for postural hyperkyphosis: a randomized clinical trial. Physiother Theory Pract. 2016;32(2):92–7. 26863146 10.3109/09593985.2015.1110739

[10] Sharma K, Chawla JK, Parasher RK. Role of dynamic neuromuscular stabilization exercises in physical rehabilitation: a systematic review. Crit Reviews™ Phys Rehabilitation Med. 2024;36(1).

[11] Plisky P, Schwartkopf-Phifer K, Huebner B, Garner MB, Bullock G. Systematic review and meta-analysis of the Y-balance test lower quarter: reliability, discriminant validity, and predictive validity. Int J Sports Phys Therapy. 2021;16(5):1190. 10.26603/001c.27634 PMC8486397 34631241

[12] Honarvar A, Mazloum V, Soleymanfallah MA. Effects of a Warm-Up injury prevention protocol on risk factors for anterior cruciate ligament injury in elite basketball players. J Mod Rehabilitation. 2025.

[13] Livanelioglu A, Kaya F, Nabiyev V, Demirkiran G, Fırat T. The validity and reliability of spinal mouse assessment of spinal curvatures in the frontal plane in pediatric adolescent idiopathic thoraco-lumbar curves. Eur Spine J. 2016;25(2):476–82. 25900295 10.1007/s00586-015-3945-7

[14] Livanelioglu A, Kaya F, Nabiyev V, Demirkiran G, Fırat T. The validity and reliability of spinal mouse assessment of spinal curvatures in the frontal plane in pediatric adolescent idiopathic thoraco-lumbar curves. Eur Spine J. 2016;25:476–82. 25900295 10.1007/s00586-015-3945-7

[15] Wu Q, Chen Y, Zhou Y, Zhang X, Huang Y, Liu R. Reliability, validity, and sensitivity of short-form 36 health survey (SF-36) in patients with sick sinus syndrome. Medicine. 2023;102(24):e33979. 37327281 10.1097/MD.0000000000033979 PMC10270486

[16] Rahimi M, Hasanpor Z, Sharifi R, Haghighi M. Effect of eight-week dynamic neuromuscular stabilization training on balance, fall risk and lower extremity strength in healthy elderly women. Stud Sport Med. 2020;12(28):107–26.

[17] Mokhtaran S, Piri H, Sheikhhoseini R, Salsali M. Comparing two corrective exercise approaches for body image and upper-quadrant posture in schoolgirls with hyperkyphosis. Sci Rep. 2025;15(1):3882. 39890832 10.1038/s41598-025-85665-0 PMC11785943

[18] Park SJ, Kim SH, Kim SH, editors. Effects of thoracic mobilization and extension exercise on thoracic alignment and shoulder function in patients with subacromial impingement syndrome: A randomized controlled pilot study. Healthcare: MDPI; 2020. 10.3390/healthcare8030316 PMC7551755 32887287

[19] Yoo W-g. Effect of thoracic stretching, thoracic extension exercise and exercises for cervical and scapular posture on thoracic kyphosis angle and upper thoracic pain. J Phys Therapy Sci. 2013;25(11):1509–10. 10.1589/jpts.25.1509 PMC3881488 24396221

[20] Jang H-J, Hughes LC, Oh D-W, Kim S-Y. Effects of corrective exercise for thoracic hyperkyphosis on posture, balance, and well-being in older women: a double-blind, group-matched design. J Geriatr Phys Ther. 2019;42(3):E17–27. 28914720 10.1519/JPT.0000000000000146

[21] Davidek P, Andel R, Kobesova A. Influence of dynamic neuromuscular stabilization approach on maximum Kayak paddling force. J Hum Kinetics. 2018;61:15. 10.1515/hukin-2017-0127 PMC5873333 29599856

[22] De Troyer A, Wilson TA. Mechanism of the increased rib cage expansion produced by the diaphragm with abdominal support. J Appl Physiol. 2015;118(8):989–95. 25678694 10.1152/japplphysiol.00016.2015

[23] Ghavipanje V, Rahimi NM, Akhlaghi F. Six weeks effects of dynamic neuromuscular stabilization (DNS) training in obese postpartum women with low back pain: A randomized controlled trial. Biol Res Nurs. 2022;24(1):106–14. 34555964 10.1177/10998004211044828

[24] Mohammad Rahimi N, Mahdavinejad R, Attarzadeh Hosseini SR, Negahban H. Efficacy of dynamic neuromuscular stabilization breathing exercises on chest mobility, trunk muscles, and thoracic kyphosis: a randomized controlled 6-Week trial. Iran Rehabilitation J. 2020;18(3):329–36.

[25] Afsari Z, Rahimi NM, Azimkhani A. Investigating the effects of dynamic neuromuscular stabilization exercises on chest Mobility, upright sitting Height, and quality of life in obese women. Phys Treatments-Specific Phys Therapy J. 2024;14(2):137–46.

[26] Jenkins HJ, Downie AS, Fernandez M, Hancock MJ. Decreasing thoracic hyperkyphosis–Which treatments are most effective? A systematic literature review and meta-analysis. Musculoskelet Sci Pract. 2021;56:102438. 34375856 10.1016/j.msksp.2021.102438

[27] Dimitrijević V, Protić-Gava B, Vinaji T, Popović N. Effects of corrective exercises on kyphotic angle reduction: A systematic review and meta-analysis. Medicinski Pregled. 2021;74(5–6):167–73.

[28] Ponzano M, Tibert N, Bansal S, Katzman W, Giangregorio L. Exercise for improving age-related hyperkyphosis: a systematic review and meta-analysis with GRADE assessment. Arch Osteoporos. 2021;16(1):140. 34546447 10.1007/s11657-021-00998-3

