# Supplementary material for: Dynamic neuromuscular stabilization versus traditional training: a randomized controlled trial comparing their effects on balance, posture, and quality of life in adolescents with increased thoracic kyphosis
Source: BMC Sports Sci Med Rehabil. 2026 Feb 2;18:172. doi: 10.1186/s13102-025-01522-7 (PMC13059478; doi:10.1186/s13102-025-01522-7)
Supplement: Supplementary file 2 — Supplementary Material 2. [file 13102_2025_1522_MOESM2_ESM.doc]

**
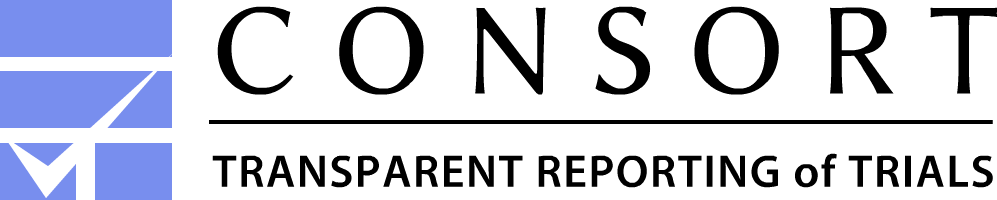
**

**CONSORT 2010 Flow Diagram**

**Allocation**

**Analysis**

**Follow-Up**

**Enrollment**

Assessed for eligibility (n= 80)

Excluded (n= 17)

♦  Not meeting inclusion criteria (n= 17)

♦  Declined to participate (n= 0)

♦  Other reasons (n= 0)

Analysed (n= 20)
♦ Excluded from analysis (absence from training sessions) (n= 1)

Lost to follow-up (absence from training sessions) (n= 1)

Discontinued intervention (give reasons) (n= 1)

Allocated to DNS group (n= 21)

♦ Received allocated intervention (n= 21)

♦ Did not receive allocated intervention (give reasons) (n= 0)

Allocated to control group (n= 21)

♦ Received allocated intervention (n= 21)

♦ Did not receive allocated intervention (give reasons) (n= 0)

Randomised (n= 63)

Allocated to CE group (n= 21)

♦ Received allocated intervention (n= 21)

♦ Did not receive allocated intervention (give reasons) (n= 0)

Lost to follow-up (n= 0)

Discontinued intervention (n= 0)

Lost to follow-up (Missed final assessments) (n= 1)

Discontinued intervention (n= 1)

Analysed (n= 20)
♦ Excluded from analysis (Withdrawal) (n= 1)

Analysed (n= 20)
♦ Excluded from analysis (Missed final assessments) (n= 1)
